# Three-Dimensional-Printed Photocatalytic Sponges Decorated with Mn-Doped ZnO Nanoparticles

**DOI:** 10.3390/ma16165672

**Published:** 2023-08-18

**Authors:** Nikolaos Rafael Vrithias, Klytaimnistra Katsara, Lampros Papoutsakis, Vassilis M. Papadakis, Zacharias Viskadourakis, Ioannis N. Remediakis, George Kenanakis

**Affiliations:** 1 Institute of Electronic Structure and Laser, Foundation for Research & Technology-Hellas, N. Plastira 100, 700 13 Heraklion, Crete, Greece; n.vrithias@iesl.forth.gr (N.R.V.);; 2 Department of Materials Science and Technology, University of Crete, 710 03 Heraklion, Crete, Greece; 3 Department of Agriculture, Hellenic Mediterranean University, Estavromenos, 714 10 Heraklion, Crete, Greece; 4 Department of Chemistry, University of Crete, 710 03 Heraklion, Crete, Greece; 5 Department of Industrial Design and Production Engineering, University of West Attica, 122 43 Athens, Greece

**Keywords:** photocatalysis, Mn-doped ZnO, nanomaterials, FDM 3D printing, liquid laundry detergent degradation

## Abstract

The present work reports on the fabrication of high-density polyethylene sponges, decorated with Mn-doped ZnO nanostructures. The sponges were developed utilizing three-dimensional printing technology, while Mn-doped ZnO nanostructures, with varying doping levels, were grown at mild temperatures. The nanostructures were fully characterized by means of scanning electron microscopy, X-ray diffraction, and Raman spectroscopy, revealing the existence of Mn doping. Moreover, their photocatalytic properties were investigated using the degradation/decolorization of a commercially available liquid laundry detergent, based on synthetic, less foaming ingredients, under UV irradiation. The Mn-doped ZnO nanostructures show better photocatalytic activity at higher doping levels. This study demonstrates that it is possible to achieve the adequate degradation of a typical detergent solution in water by means of low-cost and environmentally friendly approaches, while Mn-doped ZnO/HDPE nanostructures are good candidates for real environmental applications.

## 1. Introduction

Our planet is mostly covered with water; it is a vital element for human beings and all life on Earth, in general. If we take into account the increases in the population of our planet [1], one can easily understand the need for more clear and pure water, free from harmful substances. However, at the same time, water is polluted in many ways; this can take the form of waste water release by industrial establishments, the mixing of water with fertilizers channeled through groundwater routes [2], pharmaceutical products [3], or even detergent residuals ending up in the sewage system and in the sea, despite the typical purification treatment by municipal establishments.

Detergents have always been in wide-spread use by humans, and their use is expected to expand even more [4]. It should be noted that the development of new types of surfactants, more sustainable than existing ones, the so-called “biosurfactants”, are up to 10–12 times more expensive, and this cost makes the replacement of less sustainable types quite difficult [5].

Several treatment methods are proposed for the elimination of harmful surfactants, such as electrocoagulation [6], nanofiltration [7], sand sorption [8], and others [9]. These approaches offer a solution, but there is always the challenge of making the processes easier and more cost-effective in order to treat large amounts of wastewater and remove detergent compounds from treated water. These residues can be eliminated via oxidation methods, and advanced heterogeneous photocatalysis appears to be the most promising approach, which includes the use of inert catalysts, non-hazardous oxidants, ultraviolet light (UV) and/or visible light input [10,11,12,13,14].

Heterogeneous photocatalysis is a process that generates free radical •OH from atmospheric air, instead of O_3_ or H_2_O_2_, and significantly reduces water processing costs. This approach occurs under ambient conditions and leads to the complete decomposition of both liquid and gaseous pollutants [15,16]. The most important advantage of this process is that universal, environmentally friendly catalysts are inexpensive, non-toxic and photo-stable compared to other photocatalysts, as well as easily available and easily reusable, maintaining the same high performance in a large number of catalyst cycles. On the other hand, the main disadvantage of using photocatalysts in the form of a powder semiconductor is that it must be removed after treatment [17,18]. For this reason, international efforts have been concentrated on photocatalytic systems, in which catalysts are used in the form of film on inert substrates to eliminate powder removal [11,19]. In most cases, however, due to manufacturing technologies limitations and the potential limitation of their use in real-world applications, samples of solid catalysts such as thin films or nanostructures cannot exceed a few centimeters in total size.

In general, photocatalytic samples need to have as high a surface-to-volume ratio as possible as well as the ability to be easily reused. A pathway to achieve this is to employ 3D printing and, more specifically, the fused deposition method (FDM), one of the most commonly utilized low-cost processes due to its simplicity and the availability of machines at affordable prices, in which polymers are the usual materials used as filaments. During this process, a polymeric filament is melted through a heated nozzle and deposited onto a platform in a raster pattern to form each layer of the model, while the final 3D product is fabricated by building up successive layers [20]. Typically, FDM polymeric filaments consist of thermoplastics, such as polylactic acid (PLA), acrylonitrile butadiene styrene (ABS), etc. [20].

Although polymers are widely available, commercial FDM materials are still limited, expensive, and lack good properties, causing significant difficulties in the use of FDM technology in production [21,22]. ABS is a fairly light polymer and is therefore easier to mold into complex shapes but requires the addition of Butadiene to be chemically resistant, which is extremely toxic and harmful to humans and animals. On the other hand, PLA cannot be recycled and mixed with common polymers, it must be sorted separately and brought to a “closed composting environment”, since it otherwise contaminates the recycling stream [23,24]. Furthermore, PLA cannot be used for high temperature applications, since it has low heat resistance. At high temperatures, PLA deforms rapidly, especially under pressure, while it is typically weaker and has lower tensile strength than its counterpart, ABS [25].

In contrast to the above polymers, polyethylene (PE) and especially high-density polyethylene (HDPE) have excellent toughness, cut resistance, and wear resistance and are well-known for their good chemical resistance [26]. Furthermore, the U.S. Food and Drug Administration (FDA) has approved HDPE as a BPA-free polymer that does not leach into its contents, providing quite high guarantee for preserving drinking water quality and reducing the possibility of spillage of waste water into the environment [27].

Although there are several reports on 3D structures for novel environmental applications using a variety of polymers [28,29,30,31,32], there is no record based on HDPE filaments used in FDM technology for environmental applications and especially for the removal of residual detergents by photocatalysis.

In this work, we present the fabrication of FDM 3D-printed sponges, fabricated using pure high-density polyethylene (HDPE), decorated with state-of-the-art photocatalysts. Sponge-like geometries were chosen in order to make visible the fabrication of porous samples, suitable for filtering in water treatment systems. The combination of such sponges of various porous sizes with effective photocatalysts gives the opportunity to clean water in a single stage; different solid materials (such as sand, gravel, charcoal, etc.) can be retained and separated through filtering, while dissolved particles and germs (such as chemicals, parasites, bacteria, viruses, etc.) can be eliminated through UV-assisted photocatalysis. Titanium dioxide (TiO_2_) has been one of the most studied photocatalysts, among several materials, as it has a band gap of about 3.2 eV, high photocatalytic efficiency, chemical and optical stability, etc. [33,34]. Meanwhile, zinc oxide (ZnO) has attracted tremendous interest as an efficient photocatalytic material because of its energy level, which is comparable to the band gap of TiO_2_ [35]; electron mobility; high life expectancy; and relatively low production cost. Furthermore, compared to TiO_2_, ZnO can be fabricated in powder form [36] or as a coating on top of numerous substrates [37,38], following environmentally friendly chemical approaches. Finally, ZnO can form multipurpose nanostructures on different substrates, changing from prisms and rods [39] to wires [39], and it can absorb more light quanta [40].

The present study focuses on the decoration of 3D-printed sponge-like scaffolds with pure and Mn-doped ZnO nanostructures, designed to work as photocatalytic devices for the degradation of a commercially available liquid laundry detergent, based on synthetic, less foaming ingredients; Dixan (Henkel AG & Co. KGaA, Düsseldorf, Germany). Here, four types of ZnO-based nanomaterials containing 0.0, 1.0, 5.0, and 10.0 wt% of manganese were synthesized following an aqueous solution growth approach at 95 °C. Mn was chosen as a dopant element of ZnO in order to produce high-efficiency photocatalysts with enhanced performance due to their lower energy gap, compared to pure ZnO (0.16%) under UV light. We provide evidence that doping ZnO with an optimal amount of Mn leads to a decrease in the band gap [41], following a low-temperature, environmentally friendly approach. The structural properties of the prepared samples were studied using X-ray diffraction (XRD) and Raman spectroscopy, while their morphology and elemental composition was examined by means of scanning electron microscopy (SEM) and energy-dispersive X-ray spectroscopy (EDS). The photocatalytic properties of the nanomaterial-based 3D-printed samples were evaluated using commercially available Dixan as the target pollutant in an aqueous solution under ultraviolet (UV) light in a self-designed photocatalytic reactor.

It is envisioned that our work may provide a new approach for preparing nanosized Mn-doped ZnO photocatalysts on 3D-printed sponge-like filters, suitable for practical applications.

## 2. Experimental Part

### 2.1. Fabrication of 3D-Printed Sponges

**Figure 1:**
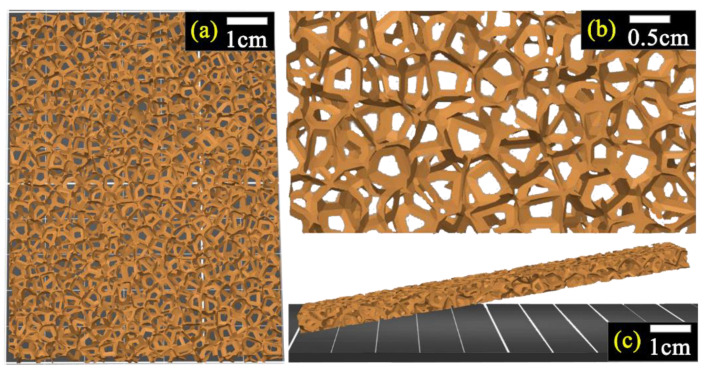
A 3D model of sponge-like samples: top view (**a**), magnification (**b**), and side view (**c**).

The drawings of sponge-shaped scaffolds, such as those pictured in Figure 1, were designed using the free online 3D modeling program “Tinkercad” (Autodesk Inc., San Francisco, CA, USA). Subsequently, a “Makerbot Replicator 2X” FDM 3D printer (MakerBot Industries, Brooklyn, NY, USA) was used for the fabrication of 3D structures/scaffolds. Commercially available HDPE filament (3D-Printerstore, Weinfelden, Switzerland) was used as spool material for the printing process. During printing, the nozzle temperature was kept at 220 °C, while the printing bed temperature was at 110 °C. Each printing layer was 0.2 mm and the filling factor was fixed at 100% in order to develop fully packed samples.

According to the above-mentioned method, sponge-like 3D-printed samples were designed, and both their geometry and dimensions are presented in Figure 1. A tetrahedral unit cell was used as a starting element and several units were put together in space to form porous structures with typical dimensions 100 mm × 70 mm × 5 mm.

A solid network of HDPE lines, with random interlinks among them, was developed. Additionally, pores/holes, with diameters ranging from ~0.2 mm to 1.0 mm to 2.0 mm, were inhomogeneously formatted, between interlinked HDPE lines. Therefore, scaffolds of sponge-like structure were successfully grown using 3D printing technology.

### 2.2. Synthesis of Photocatalytic Nanostructures—Decoration of HDPE Sponges

Mn-doped nanostructures were grown, employing the so-called aqueous solution growth on the 3D-printed sponges mentioned above [42,43,44]. An equimolar (0.01 M) aqueous solution of Zn(NO_3_)_2_·6H_2_O and C_6_H_12_N_4_ was used, while manganese (II) nitrate tetrahydrate (Mn(NO_3_)_2_·4H_2_O) (Sigma-Aldrich, Munich, Germany) served as the dopant source. The Mn molar concentration in the solution varied from 0 to 10%. First, laboratory Pyrex glass bottles with autoclavable polypropylene screw caps were filled with the solution described above. In addition, pieces of the 3D-printed sponges produced were also placed in the bottles and heated at a constant temperature of 95 °C for 5 h in a regular laboratory oven. In such a way, Mn–ZnO nanostructures were developed directly onto the sponge scaffolds. After heating, the samples were then thoroughly washed with MilliQ water (18.2 MΩ cm) and dried in air at the same temperature.

### 2.3. Characterization and Photocatalytic Experiments

#### 2.3.1. Optical and Scanning Electron Microscopy

The surface morphology of the ZnO-coated 3D-printed structures was studied using an AP-8 optical microscope (Euromex Microscopen bv., Arnhem, The Netherlands), achieving magnifications up to ×80. Furthermore, scanning electron microscopy (SEM) images were obtained using a field emission scanning electron microscope (FE-SEM, JEOL JSM-7000F; JEOL Ltd., Tokyo, Japan). Microscopy characterization was performed in high vacuum (HV) using ~10 nm Au/Pd-coated samples.

#### 2.3.2. X-ray Diffraction X-ray

X-ray diffraction (XRD) was used to determine the crystal structure of the prepared Mn-doped ZnO-coated 3D-printed structures using a Bruker D8 Advance Diffractometer (Bruker AXS Advanced X-ray Solutions GmbH, Karlsruhe, Germany). The device was equipped with TWIN-TWIN technology and a Cu-sealed tube source (wavelength 1.54 Å). The diffraction data were recorded in theta–2theta mode in the range 30–70 degrees for 2theta, with step 0.02 degrees and speed 3 s/step, while the samples were placed on a Si wafer to minimize background noise.

#### 2.3.3. Raman Spectroscopy Studies

Raman spectroscopy measurements were performed at room temperature using a Horiba LabRAM HR Evolution confocal microspectrometer (HORIBA FRANCE SAS, Longjumeau, France), via backscattering geometry (180°), equipped with an air-cooled solid state laser (HORIBA FRANCE SAS, Longjumeau, France), operating at 532 nm with 90 mW output power. Each sample was placed on a stainless steel microscope slide and the laser beam was focused on the samples, through a 25% ND filter, using a 10× Olympus MPlan microscope objective (OLYMPUS Corporation, Tokyo, Japan) (numerical aperture of 0.25), resulting in a ~11 mW power on each sample. Raman spectra over the 200–1200 cm^−1^ wavenumber range (with an exposure time of 5 s and 3 accumulations) were collected by a Peltier cooled CCD (1024 × 256 pixels) detector at −60 °C. The size of the laser spot, referring to the microscope resolution, was approximately 2.596 μm, resulting in a Raman spectral resolution of ~2 cm^−1^, achieved thanks to a 600 grooves/mm grating and an 800 mm focal length. Test measurements carried out using different optical configuration, exposure time, beam power, and accumulations to obtain sufficiently informative spectra using a confocal hole of 100 μm but avoiding alteration of the sample, while the high spatial resolution allowed us to carefully verify the sample homogeneity. The wavelength scale was calibrated using a Silicon standard (Silchem Handelsgesellschaft mbH, Freiberg, Germany) (520.7 cm^−1^) and the acquired spectra were compared with published scientific data and reference databases, such as Horiba LabSpec 6 (HORIBA FRANCE SAS, Longjumeau, France).

#### 2.3.4. Photocatalytic Efficiency Measurements

The photocatalytic activity of the 3D-printed samples was studied by means of the reduction of an aqueous solution of Dixan (Henkel AG & Co. KGaA, Düsseldorf, Germany), obtained from a local supermarket in Greece. First, 1 mL of commercially available laundry detergent Dixan (code “Ocean”) was diluted in 12 mL of tap water (~8.33% *v*/*v*). Subsequently, Mn–ZnO-decorated 3D-printed samples (1 × 1 cm^2^) were placed in a custom-made quartz cell, and the whole setup (cell + solution + sample) was illuminated for up to 60 min using a UV lamp centered at 365 nm (Philips HPK 125 W) (msscientific Chromatographie-Handel GmbH, Berlin, Germany) with a light intensity of ~6.0 mW/cm^2^. The concentration of Dixan (decolorization) was monitored via UV–Vis spectroscopy in absorption mode (absorption at λmax, 353 nm), using a “BIOBASE BK-D590” Double Beam Scanning UV/VIS Spectrophotometer (BIOBASE, Jinan, Shandong, China), over a wavelength range of 190–1100 nm, using a 1200 lines/mm grating. In such a way, UV–Vis absorption data were collected at 0, 10, 20, 30, 40, 50, and 60 min, while the quantification of Dixan degradation (and hence the remained Dixan concentration) was estimated via the calculation of the area below the main absorption peak in the range of 280–400 nm. Photolysis (blank experiments without a catalyst), as well as Dixan adsorption experiments in dark, were performed. To ensure repeatability, the photocatalysis experiments were performed at least five times.

## 3. Results and Discussion

**Figure 2:**
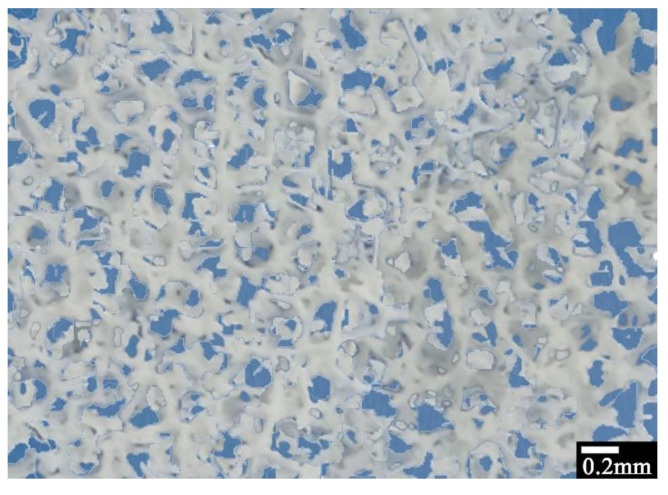
Optical microscopy picture of 3D-printed sponges.

Figure 2 shows a typical optical microscopy picture of the 3D-printed sponges. It can be seen that the 3D-printed sponges consist of an HDPE network with pores/holes of the order of 0.05–0.2 mm along with bigger ones (>1.0 mm), in agreement with the 3D model in Figure 1.

**Figure 3:**
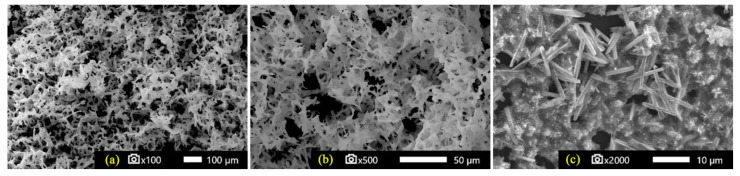
SEM images of 3D-printed sponges, prior to (**a**,**b**) and after their decoration with Mn–ZnO (**c**).

Figure 3 depicts SEM micrographs of the 3D-printed sponges, before (Figure 3a,b) and after their decoration with Mn–ZnO (Figure 3c). Highly porous HDPE structures were observed, which is typical for their sponge-like nature. The diameter of the pores/hole layers was in the range of 50–200 μm. Compared to Figure 1, it can be concluded that 3D-printed sponges include small (50–200 µm) as well large (1.0–2.0 mm) pores/holes, which are inhomogeneously distributed, resulting in a bimodal pore/hole mixture. Figure 3c shows typical Mn–ZnO nanostructures (for 10% Mn doping), which were grown on the sponges. It is worth noting that Mn-doping did not influence the geometry of the nanostructures. The grown structures consist of nanorods with diameters ranging from 0.8 to 1.0 μm and a length of ∼8–10μm, and they are similar to the un-doped ZnO nanostructures reported elsewhere [45]. Therefore, the Mn–ZnO nanostructure decoration of the 3D-printed HDPE sponges was successfully accomplished.

**Figure 4:**
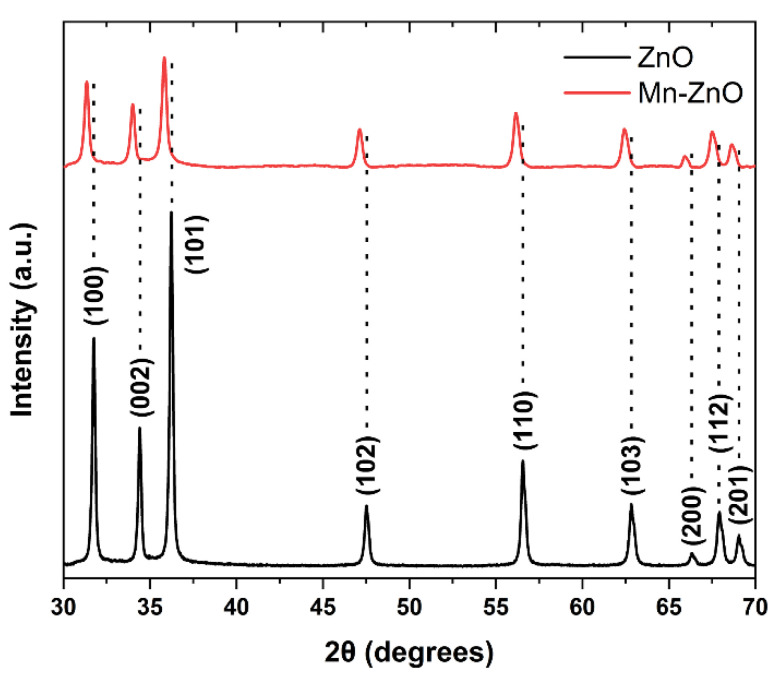
XRD pattern of ZnO (black) and Mn-doped ZnO (red) nanostructures deposited on 3D-printed sponges for 5 h at 95 °C.

Figure 4 presents the XRD pattern of the typical samples prepared (i.e., pure ZnO, black curve; 10% Mn–ZnO, red curve). The XRD pattern of the pure ZnO sample is consistent with the hexagonal wurtzite (JCPDS No. 80-0075). There are no obvious MnO diffraction peaks, indicating that Mn^2+^ ions are incorporated into the crystal structure of ZnO, which is likely coupled with the low concentration of Mn(II) on the surface of the ZnO [46,47,48]. A closer look of the XRD peak positions indicates a slight shifting of the diffraction peaks in Mn-doped ZnO, compared to that of pure ZnO, toward lower 2θ values. According to Bragg’s law (i.e., *2d* sin* θ = nλ*), since the Zn^2+^ ion radius (0.74 Å) is smaller than that of the Mn^2+^ (i.e., 0.8 Å) [49,50], the Mn^2+^ doping thus increases the lattice constant [51]. As a result, the XRD peaks shift slightly to lower angles [50,52,53], as indicated in Figure 4.

**Figure 5:**
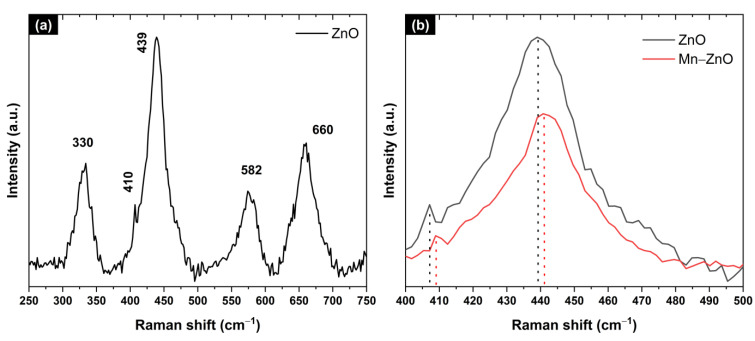
(**a**) Raman spectra of ZnO (black) and 10% Mn-doped ZnO (red) nanostructures deposited on 3D-printed sponges for 5 h. In (**b**), one can see a zoom plot of E_2_ (high) and A_1_ (TO).

Raman spectra of pure and Mn-doped ZnO nanostructures were investigated in the range 250–750 cm^−1^, as shown in Figure 5.

We can observe common phonon modes for pure ZnO and Mn-doped nanostructures, typical for a wurtzite structure with a C_6v_ point group symmetry, centered at around 330, 407, 439, 582, and 660 cm^−1^, and are attributed to the processes E_2_ (high), A_1_ (TO), E_1_ (TO), E_2_ (high), 1LO, and TA + LO, respectively [54,55,56,57,58]. The 1LO mode corresponds to the superimposition of A_1_ (LO) and E_1_ (LO), which are characteristic of the presence of randomly-oriented ZnO crystals, while the strong peak at 439 cm^−1^ is associated with the high-frequency E_2_ mode of oxygen atoms and indicates a ZnO wurtzite hexagonal phase [55,59], which was confirmed using XRD. Finally, if one looks carefully at Figure 5a and the zoom plot of Figure 5b, one can notice a shift in the E_2_ (high) and A_1_ (TO) modes towards higher frequencies, i.e., from 439.01 to 440.78 cm^−1^ and from 407.13 to 418.91 cm^−1^, respectively, due to Mn-doping [60,61,62].

After their optical and structural characterization, the Mn–ZnO-decorated sponges were studied in terms of their photocatalytic properties. In particular, the reduction/decolorization of liquid laundry detergent in aqueous solution was evaluated via the photocatalytic activity of Mn-doped ZnO nanostructures under UV–A light. The photolysis of the detergent in the absence of any photocatalyst was negligible, indicating the indispensability of the catalysts. The detergent solution gradually faded as the photocatalytic process was carried out, which obviously indicated that the detergent concentration decreased. This phenomenon can be attributed to the destruction of the entire molecular structure or to the destruction of the chromophore. Furthermore, to eliminate the possibility of detergent removal through adsorption to the catalysts, the samples were placed at the bottom of the reactor in dark conditions and were left in contact with the detergent solution for 30 min, during which time the equilibrium of adsorption–desorption was achieved. In all cases, removal was insignificant (less than 3%), indicating that the reduction of the Dixan solution should be attributed to a pure photocatalytic procedure.

An example of the typical decrease in detergent concentration (8.33% *v*/*v*) in the presence of ZnO/Mn–ZnO nanostructures under UV–A light irradiation is presented in Supplementary Figure S1, indicating that 30 min are sufficient for the adequate degradation of the detergent’s solution. The quantification of detergent degradation (and hence the remaining Dixan concentration) was estimated through the calculation of the area below the main absorption peak in the range of 280–400 nm. The decrease in detergent concentration (decolorization) for pure and Mn-doped ZnO nanostructures under UV–A light was compared to photolysis (no catalyst present) for ~60 min of irradiation, indicating that the photolysis of Dixan was almost negligible.

In addition, the apparent rate constants (*k*) were calculated as the basic kinetic parameters for the comparison of photocatalytic activities, which were fitted using the following equation:

*ln* (*C_t_*/*C*_0_) = −*kt* (1)

where *k* is the apparent rate constant, *C_t_* is the concentration of Dixan, and *C*_0_ is its initial concentration.

The apparent rate *k* follows a good linear fit of Equation (1) (Supplementary Figure S2), indicating that the degradation/decolorization of the detergent follows first-order kinetics. Furthermore, it should be noted that the adjusted R-square statistic for the calculation of the apparent rate constants varied from 0.884 to 0.990, indicating that the model used for the determination of the apparent rate constant (*k*) is adequate.

**Figure 6:**
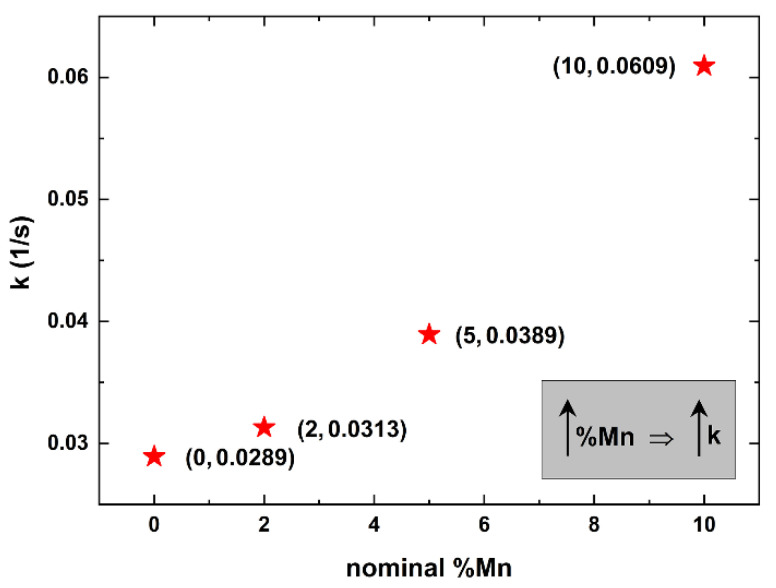
Apparent rate constants for the degradation/decolorization of Dixan using pure and Mn-doped ZnO on 3D-printed sponges, as a functionally nominal %Mn.

Figure 6 indicates the apparent rate constants for detergent using pure and Mn-doped ZnO deposited on 3D-printed sponges.

The apparent rate constant for pure ZnO (nominal %Mn = 0) is ~0.0289, considerably lower than the ones of the Mn-doped samples (nominal %Mn = 2–10). Furthermore, one can easily see that as the nominal %Mn increases, the degradation/decolorization of the detergent becomes faster, reaching a maximum k value of 0.0609.

The above finding indicates that Mn-doping was indeed successful, as indicated in Figure 4 and Figure 5. As we already stated, Mn-doping did not influence the geometry of the nanostructures. As a result, the photocatalytic enhancement of the Mn-doped samples over the pure ZnO ones is probably be due to Mn, since Mn doping decreases the energy gap of ZnO from ~3.4 eV [54] to ~3.0 eV for 10% Mn [63], thus making Mn-doped ZnO a better candidate for photocatalytic applications [64,65,66].

**Figure 7:**
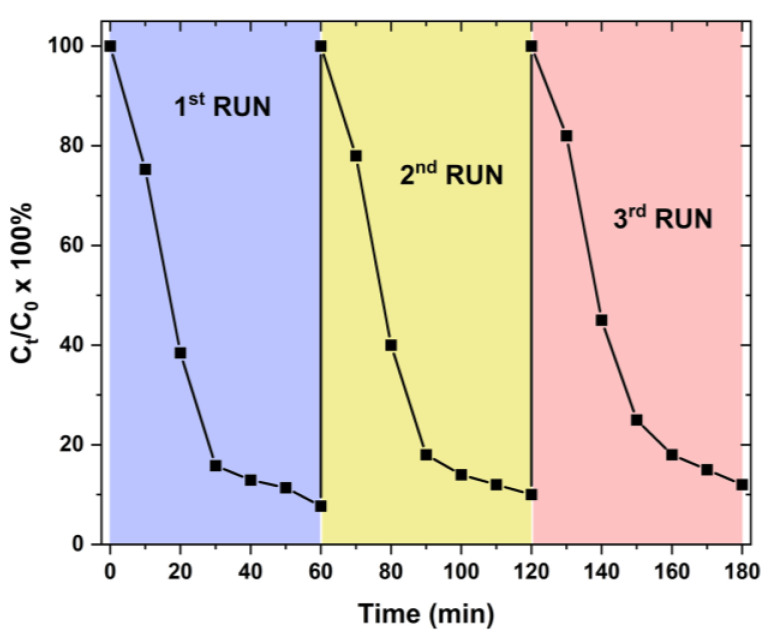
The % of degradation/decolorization of Dixan over 10% Mn-doped ZnO samples deposited on 3D-printed sponges over three runs of 60 min irradiation each.

In order to verify the use of our samples for real-life applications, their stability was tested under the same conditions; we recovered each sample and tested their efficiency over three runs. Figure 7 depicts the re-use of the 10% Mn-doped ZnO samples deposited on 3D-printed sponges over three runs.

It is evident that the 10% Mn-doped ZnO samples on 3D printed sponges can be successfully used at least three times for the photodegradation (decolorization) of Dixan, reaching an efficiency of ~88% at the end of the third run.

## 4. Conclusions

Mn-doped ZnO nanostructures with hexagonal crystal structures were grown on 3D-printed HDPE sponges at mild temperatures of as low as 95 °C, with a dopant nominal concentration ranging from 0.0 to 10.0%. The samples were characterized via SEM, XRD, and Raman spectroscopy, revealing the existence of manganese at the zinc sites in the Mn-doped ZnO nanostructures. The Mn-doped ZnO nanostructured samples display better photocatalytic activity against a commercially available liquid laundry detergent, based on synthetic, less foaming ingredients, under UV irradiation when the doping level is higher (10 at%). This better photocatalytic efficiency at higher doping levels is due to the interstitial incorporation of Mn and Zn, as evidenced by the X-ray diffraction (XRD) and Raman results.

## Data Availability

Data are available upon request from the corresponding author.

## Supplementary Materials

The following supporting information can be downloaded at: https://www.mdpi.com/article/10.3390/ma16165672/s1, Figure S1: Typical absorption spectra of photocatalytic degradation of 8.33% *v*/*v* Dixan aqueous solution in the presence of ZnO/Mn–ZnO nanostructures under UV–A light irradiation; Figure S2: Apparent rate constants (k) of Dixan degradation using Mn-doped ZnO nanostructures deposited on 3D-printed sponges.
