# Supplementary material for: Three-Dimensional-Printed Photocatalytic Sponges Decorated with Mn-Doped ZnO Nanoparticles
Source: Materials (Basel). 2023 Aug 18;16(16):5672. doi: 10.3390/ma16165672 (PMC10456673; doi:10.3390/ma16165672)
Supplement: Supplementary file 1 [file materials-16-05672-s001.zip › materials-2527784-supplementary.pdf]

Supplementary Material

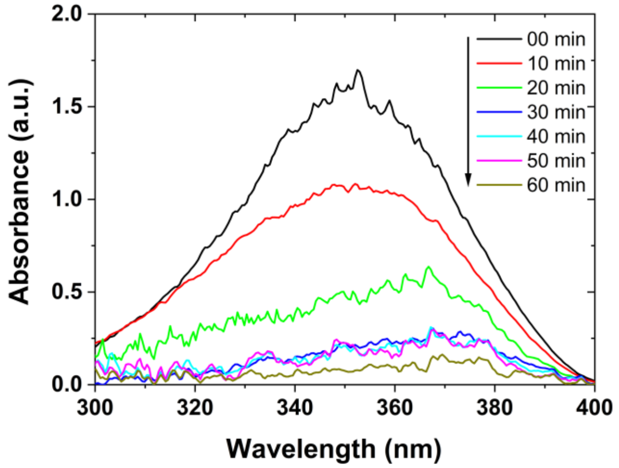

Figure S1. Typical absorption spectra of photocatalytic degradation of 8.33% v/v Dixan aqueous solution in the presence of ZnO/Mn-ZnO nanostructures under UV-A light irradiation.

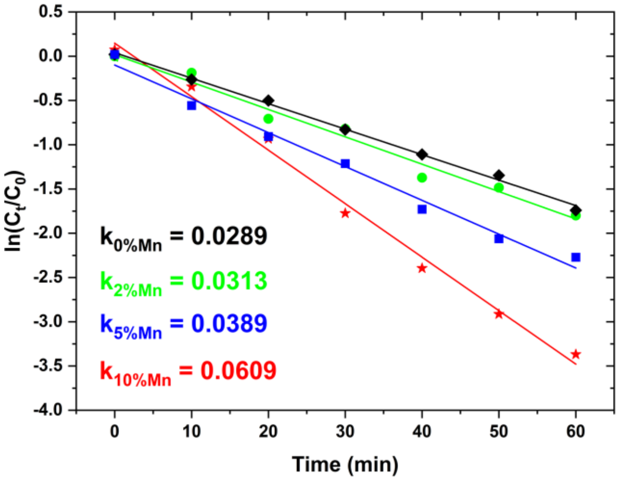

Figure S2. Apparent rate constants (k) of Dixan degradation using Mn-doped ZnO nanostructures deposited on 3D printed sponges.
